# Age-Related Differences for Cardiorespiratory Fitness Improvement in Patients Undergoing Cardiac Rehabilitation

**DOI:** 10.3389/fcvm.2022.872757

**Published:** 2022-04-14

**Authors:** Jenna L. Taylor, Jose R. Medina-Inojosa, Audry Chacin-Suarez, Joshua R. Smith, Ray W. Squires, Randal J. Thomas, Bruce D. Johnson, Thomas P. Olson, Amanda R. Bonikowske

**Affiliations:** ^1^Division of Preventive Cardiology, Department of Cardiovascular Medicine, Mayo Clinic, Rochester, MN, United States; ^2^Human Integrative and Environmental Physiology Laboratory, Department of Cardiovascular Medicine, Mayo Clinic, Rochester, MN, United States; ^3^Division of Epidemiology, Department of Quantitative Health Sciences, Mayo Clinic, Rochester, MN, United States

**Keywords:** peak oxygen uptake (VO_2_), exercise capacity, older adult, peak VO_2_ responder, peak VO_2_ non-responder, exercise training

## Abstract

**Objective:**

We investigated age-related differences for peak oxygen uptake (peak VO_2_) improvement with exercise training during cardiac rehabilitation (CR).

**Patients and Methods:**

This was a retrospective cohort study of the Mayo Clinic Rochester CR program including adult patients who attended CR (≥1 session) for any eligible indication between 1999 and 2017 and who had a cardiopulmonary exercise test pre and post CR with VO_2_ data (peak respiratory exchange ratio ≥1.0). Younger (20–49 yrs), midlife (50–64 yrs), and older adults (≥65 yrs) were compared using ANOVA for delta and percent change in peak VO_2_; and percentage of peak VO_2_ responders (>0% change).

**Results:**

708 patients (age: 60.8 ± 12.1 years; 24% female) met inclusion criteria. Delta and percent change in peak VO_2_ was lower for older adults (1.6 ± 3.2 mL.kg.min^−1^; 12 ± 27%) compared with younger (3.7 ± 4.0 mL.kg.min^−1^, *p* < 0.001; 23 ± 28%, *p* = 0.002) and midlife adults (2.8 ± 3.8 mL.kg.min^−1^, *p* < 0.001; 17 ± 28%, *p* = 0.04). For midlife, delta change, but not percent change in peak VO_2_ was significantly lower (*p* = 0.02) compared with younger. Percentage of responders was only different between older and younger (72 vs. 86%; *p* = 0.008). Sensitivity analyses in non-surgical patients showed similar differences for delta change, and differences in percent change remained significant between older and younger adults (10 ± 20% vs. 16 ± 18%; *p* = 0.04).

**Conclusions:**

In CR patients, older adults had lower improvement in cardiorespiratory fitness than younger and midlife adults. While excluding surgical patients reduced age-related differences, older adults still had lower cardiorespiratory fitness improvement during CR. These findings may have implications for individualizing CR programming in aging populations to reduce future cardiovascular risk.

## Introduction

Early outpatient (phase II) cardiac rehabilitation (CR) is internationally recognized as a class 1A recommendation for patients following a cardiovascular-related event or procedure (1, 2). CR is comprehensive secondary prevention encompassing multifaceted strategies to optimize cardiovascular risk reduction, foster healthy behaviors, and promote an active lifestyle (1). While CR originated primarily for middle-aged patients with coronary artery disease, the aging spectrum for cardiac rehabilitation has broadened over time with a range of eligible diagnoses and applications. In particular, benefits for aging populations include improvements in survival, exercise capacity, frailty, body composition, quality of life, symptom management, cognition, and socialization (3, 4).

Exercise training is an integral component of CR programs (1, 2), and exerts a multi-system effect on improving cardiovascular health (5). One of the most important benefits of exercise training in CR is the improvement in cardiorespiratory fitness, referred to as peak oxygen uptake (peak VO_2_) when directly measured using gas exchange analysis (6). Peak VO_2_ reflects an integrated ability to transport oxygen around the body, encompassing pulmonary function (ventilation and diffusion), right and left ventricular function (systolic and diastolic), ventricular-arterial coupling, vascular function (to accommodate and efficiently transport blood), and the ability of muscle cells to receive and use oxygen for aerobic energy production as well as communicating metabolic demands to the cardiovascular control center (7). Cardiorespiratory fitness is a strong predictor of cardiovascular events and all-cause mortality (6, 7), and has been shown to improve risk classification beyond traditional risk factors (7). Furthermore, changes in peak VO_2_ during CR are highly predictive of future readmissions for cardiovascular disease and all-cause mortality, with each 1 ml.kg.min^−1^ increase in peak VO_2_ associated with a 21% reduction in cardiovascular events and a 13% reduction in all-cause mortality (8).

It is well-established that cardiorespiratory fitness declines with increasing age (9–11), however the influence of age on the trainability of cardiorespiratory fitness is less clear, with evidence suggesting either little or no difference (12–14) or a reduction (15, 16) in trainability with increasing age. There are limited data evaluating the influence of age on cardiorespiratory fitness response during a CR program, particularly using directly measured peak VO_2_. Banks et al. investigated percent change in peak VO_2_ during cardiac rehabilitation across age decades, finding those in the younger age categories (<50 years), tended to show a greater percent change in peak VO_2_ than adults in each other age decade (16). However, this study excluded patients that did not respond to CR with increases in peak VO_2_. Therefore, it is unclear how non-responders may influence the age-related differences in peak VO_2_ improvements in a CR population.

The purpose of our study was to investigate age-related differences in peak VO_2_ response to an exercise-based CR program. We hypothesized that improvements in peak VO_2_ during CR are attenuated with increasing age. And furthermore, that with increasing age there will be a lower proportion of patients who are peak VO_2_ responders (i.e., elicit a change in peak VO_2_ > 0%) during CR.

## Methods

### Study Population

This was a historical cohort of consecutive patients residing in Olmsted County, Minnesota, who underwent CR at Mayo Clinic Rochester between June 1999 and July 2017. Patients enrolled in CR were included if they were 18 years or older and had completed a cardiopulmonary exercise test (CPET) pre and post cardiac rehabilitation, with peak VO_2_ data and peak respiratory exchange ratio ≥1.0 (indicative of sufficient exertion) (17). If, over follow-up, patients were enrolled in cardiac rehabilitation multiple times, only their earliest program enrollment was included. The CPET performed closest to the cardiac rehabilitation enrollment and discharge was included in the study, including CPETs for diagnostic purposes. This study was approved by the Mayo Clinic and Olmsted Medical Center (whose patients attended CR at Mayo Clinic) Institutional Review Boards and, per MN statute. Only patients who had provided authorization to use their medical records for medical research were included.

### Cardiac Rehabilitation Program

The CR program at Mayo Clinic in Rochester, Minnesota is based on guidelines from the American Association of Cardiovascular and Pulmonary Rehabilitation, American Heart Association, and American College of Cardiology. It is a comprehensive secondary prevention program that encompasses core components including exercise training and physical activity counseling, weight management and nutritional counseling, cardiovascular risk factor management, medication management and adherence, stress management, depressive symptom management, and social support networking. All patients are prescribed 36 exercise sessions which were typically completed at a frequency of two to three times per week over 12–18 weeks. Each exercise session consisted of 20–45 min of structured aerobic exercise and 10–15 min of resistance training, following an individualized prescription from an exercise physiologist depending on physical capabilities and/or limitations. Exercise was commonly prescribed at a moderate intensity using ratings of perceived exertion of 12–14 on the 6–20 Borg Scale (18). On days of the week with no supervised CR sessions, patients were encouraged to engage in physical activity independently for at least 30 min consistent with the prescribed exercise program.

### CPET Data Collection

Symptom-limited CPETs were conducted by trained exercise physiologists under the supervision of a cardiologist, using institutionally derived incremental exercise protocols for treadmill or cycle ergometer (19). Cardiac medications were not withheld prior to CPET. A gas exchange metabolic system (MGC Diagnostics, St Paul, Minnesota, USA) was used to quantify VO_2_, carbon dioxide production (VCO_2_), respiratory exchange ratio (RER), ventilation (V_E_), and ventilatory equivalent for VCO_2_ (VE/VCO_2_) according to the American Heart Association Exercise Standards Statement for Testing and Training (20). Peak values were obtained during the final minute of exercise. Predicted peak VO_2_ was calculated using Wasserman-Hansen and FRIEND registry prediction equations (21, 22). We further calculated the percentage of predicted peak VO_2_ achieved using our directly measured peak VO_2_. As a measure of exercise capacity, peak workload was converted to estimated metabolic equivalents (METs) using FRIEND registry equations for treadmill and cycle (23, 24). Peak oxygen pulse (mL/beat) was calculated as peak VO_2_ (in mL/min) divided by peak heart rate (beats/min) (25). The CPET data were extracted electronically from an institutional registry. To evaluate accuracy, a fraction of the data were reviewed by a clinical investigator (J.L.T). If relevant data were missing, electronic medical records were reviewed, and data were manually extracted.

### Clinical Data Collection

Patient demographic and clinical characteristics were extracted electronically using resources of the Rochester Epidemiology Project (REP) (26), a record linkage system that captures data on health conditions (diagnoses, procedures, and other vital information) for all Olmsted County residents who receive medical care at Mayo Clinic or Olmsted Medical Center and its allied health care centers (27, 28). Clinical variables were operationalized as per the International Classification of Diseases-9 and 10 (ICD-9 and−10) (29). This data extraction approach has been previously validated and reported (30, 31). A random sample of these variables was reviewed in duplicate for validation (A.C.S, J.M.I), with an excellent inter-observer agreement (all κ > 0.85).

### Statistical Analysis

The overarching aim of this analysis was to evaluate possible age-related differences in peak VO_2_ response from participation in an exercise-based CR program. To achieve this, differences in patient demographics and exercise testing outcomes were compared among different age groups across the lifespan (older adults ≥65 years; midlife adults 50–64 yrs; younger adults 20–49 yrs) (32, 33) and age decades. Groups were initially compared using analysis of variance (ANOVA) for continuous variables and Pearson's chi-squared for categorical data. Continuous data are reported as mean values and standard deviations (SD), and categorical data as frequencies and percentages. Age-group differences for delta and percent change in relative peak VO_2_ and peak workload from pre to post CR, as well as the proportion of patients classified as peak VO_2_ responders (i.e., >0% change in peak VO_2_) were also compared with ANOVA. Pre-planned *post-hoc* comparisons were conducted with pairwise *t*-test and Bonferroni correction for multiple comparisons. Due to the variation between age groups in surgical CR indications, we performed sensitivity analyses excluding patients with surgical indications to evaluate whether this influenced our results. Furthermore, we used linear regression to determine whether female sex influenced the delta or percent change in relative peak VO_2_, within age groups, with data reported as unstandardized estimate (95% confidence interval). We used logistic regression to determine whether female sex influenced the likelihood of a peak VO_2_ response within age groups, with data reported as odds ratio (95% confidence interval). A *p*-value <0.05 was considered statistically significant unless otherwise stated. All analyses were performed using BlueSky Statistics software v. 7.10 (Bluesky Statistics LLC, Chicago, IL, USA).

## Results

### Patient Demographics

A total of 708 patients met the inclusion criteria for this study. Patient demographic characteristics are outlined by age group in Table 1 and age decade in Supplementary Table 1. On average, 24% of the study population were female, with the highest proportion of females in the younger adult group. With increasing age, there was a higher proportion of patients with CR indication for percutaneous coronary intervention (PCI) (*p* < 0.001) and a lower proportion of patients with surgical CR indications (*p* = 0.002), specifically related to heart transplant (*p* < 0.001). The proportion of patients with comorbidities increased with age (*p* < 0.05) with the exception of chronic heart failure, chronic kidney disease, and smoking, which were not different between groups (*p* > 0.05). Similarly, the proportion of patients prescribed standard cardiac medications increased with age (*p* < 0.05), with the exception of calcium channel blocker and anticoagulant classes, which were not different between groups (*p* > 0.05).

**Table 1:** Patient demographics by age group.

**Demographic variable**	**Total** **(*n* = 708)**	**Younger adults** **(*n* = 115)**	**Midlife adults** **(*n* = 324)**	**Older adults** **(*n* = 269)**	***p*-value**
Age (years)	60.8 ± 12.1	41.2 ± 7.5	58.1 ± 4.3	72.5 ± 5.1	<0.001
Female	167 (24%)	40 (35%)	67 (21%)	60 (22%)	0.008
Body mass index (kg.m^−2^)	29.5 ± 5.3	29.3 ± 6.2	29.8 ± 5.5	29.2 ± 4.6	0.437
**Indication for cardiac rehabilitation**					
Acute coronary syndrome	196 (28%)	27 (23%)	90 (28%)	79 (29%)	0.497
PCI	223 (31%)	18 (16%)	105 (32%)	100 (37%)	<0.001
Surgical	173 (24%)	41 (36%)	82 (25%)	50 (19%)	0.002
Heart transplant	100 (14%)	30 (26%)	56 (17%)	14 (5%)	<0.001
CABG	36 (5%)	4 (3%)	15 (5%)	17 (6%)	0.448
Valve	33 (5%)	6 (5%)	8 (2%)	19 (7%)	0.029
Other^‡^	115 (16%)	29 (25%)	46 (14%)	50 (19%)	0.017
**Co-morbidities**					
Myocardial infarction	260 (37%)	32 (28%)	110 (34%)	118 (44%)	0.004
Hypertension	560 (79%)	61 (53%)	253 (78%)	246 (91%)	<0.001
Chronic heart failure	248 (35%)	48 (42%)	110 (34%)	90 (33%)	0.255
Dyslipidemia	642 (91%)	83 (72%)	301 (93%)	258 (96%)	<0.001
Arrhythmia	561 (79%)	78 (68%)	261 (81%)	222 (83%)	0.004
Stroke	153 (22%)	15 (13%)	53 (16%)	85 (32%)	<0.001
Chronic kidney disease	217 (31%)	27 (23%)	102 (31%)	88 (33%)	0.18
COPD	288 (41%)	31 (27%)	135 (42%)	122 (45%)	0.003
Diabetes	423 (60%)	45 (39%)	199 (61%)	179 (67%)	<0.001
Peripheral artery disease	97 (14%)	5 (4%)	34 (10%)	58 (22%)	<0.001
Smoking	451 (64%)	64 (56%)	209 (65%)	178 (66%)	0.134
**Medications**					
β-blocker	550 (78%)	80 (70%)	243 (75%)	227 (84%)	0.002
ACE Inhibitor or ARB	389 (55%)	51 (44%)	170 (52%)	168 (62%)	0.002
Calcium channel blocker	179 (25%)	23 (20%)	75 (23%)	81 (30%)	0.055
Diuretics	305 (43%)	39 (34%)	118 (36%)	148 (55%)	<0.001
Salicylates or anti-platelet	501 (71%)	66 (57%)	224 (69%)	211 (78%)	<0.001
Anticoagulant	139 (20%)	27 (23%)	48 (15%)	64 (24%)	0.012
Cholesterol lowering	566 (80%)	69 (60%)	264 (81%)	233 (87%)	<0.001

*Age groups: younger adults 20–49 yrs; Midlife adults 50–64 yrs; Older adults ≥65 yrs.*

*Continuous data are presented as mean ± SD and categorical data presented as n (%). p-values are reported for trend across group comparisons.*

*PCI, percutaneous coronary intervention; CABG, coronary artery bypass graft surgery; COPD, chronic obstructive pulmonary disease; ACE, angiotensin-converting enzyme; ARB, angiotensin II receptor blocker.*

‡ *Breakdown of other category includes, stable angina (4%), heart failure (2%), dyspnea (<1%), sudden cardiac death (<1%), peripheral artery disease (<1%), pericarditis or myocarditis (<1%), or non-specified cardiac event (8%)*.

### Baseline Exercise Testing Variables

Baseline CPET characteristics are presented by age group in Table 2 and age decade in Supplementary Table 2. With increasing age, patients had a lower resting HR and peak exercise HR, and a higher systolic blood pressure at rest and peak exercise. Baseline cardiorespiratory fitness (absolute and relative peak VO_2_) and peak workload (in estimated METs) was similar between younger and midlife adults, but lower in older adults. In contrast, younger adults achieved a lower percentage of predicted peak VO_2_ compared with both midlife and older adults. On average, ratings of perceived exertion (RPE) and RER were indicative of maximal exertion (RPE: 18.2 ± 0.9; RER: 1.19 ± 0.09), and there were no differences between groups. Sensitivity analyses, which excluded surgical patients, produced similar trends and statistical results for baseline exercise variables (Table 3). On average, baseline CPET was 6.8 ± 13.5 weeks prior to cardiac rehabilitation commencement, which reduced to 3.5 ± 10.2 weeks when surgical patients were excluded from analysis.

**Table 2:** Exercise testing and cardiac rehabilitation variables by age group.

**Outcome variable**	**Total** **(*n* = 708)**	**Younger adults** **(*n* = 115)**	**Midlife adults** **(*n* = 324)**	**Older adults** **(*n* = 269)**	***p*-value**
**Baseline CPET variables**					
CPET timing prior to program commencement (weeks)	6.8 ± 13.5	10.9 ± 16.5	7.0 ± 13.5	4.7 ± 11.5	<0.001
Resting HR (beat.min^−1^)	68 ± 12	72 ± 13	69 ± 11	66 ± 11	<0.001^a, b, c^
Resting SBP (mmHg)	115 ± 20	106 ± 18	112 ± 18	122 ± 20	<0.001^a, b, c^
Resting DBP (mmHg)	70 ± 12	68 ± 15	71 ± 11	70 ± 11	0.038^a^
Peak exercise HR (beat.min^−1^)	127 ± 23	135 ± 27	129 ± 23	120 ± 20	<0.001^a, b, c^
Peak exercise SBP (mmHg)	153 ± 35	141 ± 37	154 ± 38	158 ± 30	<0.001^a, b^
Peak exercise DBP (mmHg)	66 ± 17	62 ± 18	66 ± 17	66 ± 15	0.066
Peak exercise RPE	18.2 ± 0.9	18.2 ± 0.8	18.2 ± 1.0	18.2 ± 0.8	0.828
Peak exercise RER	1.19 ± 0.09	1.20 ± 0.11	1.19 ± 0.09	1.18 ± 0.09	0.177
Peak workload (estimated METs)	6.8 ± 1.6	7.0 ± 1.8	7.0 ± 1.7	6.4 ± 1.4	<0.001^b, c^
Peak absolute VO_2_ (L.min^−1^)	1.76 ± 0.66	1.85 ± 0.79	1.90 ± 0.69	1.56 ± 0.48	<0.001^b, c^
Peak relative VO_2_ (mL.kg.min^−1^)	20.0 ± 6.7	21.0 ± 8.1	21.1 ± 7.1	18.2 ± 4.9	<0.001^b, c^
% predicted peak VO_2_ (Wasserman-Hansen)	75 ± 22	63 ±21	76 ± 24	80 ± 19	<0.001^a, b^
% predicted peak VO_2_ (FRIEND-registry)	79 ± 33	67 ± 26	81 ± 38	82 ± 27	<0.001^a, b^
Peak oxygen pulse (mL.beat^−1^)	13.8 ± 4.2	13.4 ± 4.8	14.5 ± 4.3	13.1 ± 3.8	<0.001^a, b, c^
**Post CPET and CR program variables**					
CPET timing following program commencement (weeks)	14.9 ± 10.7	14.9 ± 10.7	16.3 ± 10.2	15.7 ± 9.3	0.367
CR program duration (weeks)	15.5 ± 10.1	12.9 ± 9.0	15.2 ± 11.0	16.9 ± 9.0	0.001^b^
CR program exercise sessions	27.0 ± 12.7	23.9 ± 13.5	25.5 ± 13.4	30.2 ± 10.9	<0.001^b, c^
Delta change in peak relative VO_2_ (mL.kg.min^−1^)	2.5 ± 3.7	3.7 ± 4.0	2.8 ± 3.8	1.6 ± 3.2	<0.001^a, b, c^
Percent change in peak relative VO_2_ (%)	16 ± 28	23 ± 28	17 ± 28	12 ± 27	0.001^b, c^
Delta change in peak workload (METs)	0.7 ± 1.1	1.0 ± 1.2	0.8 ± 1.1	0.6 ± 1.1	0.007^b^
Percent change in peak workload (%)	14 ± 24	17 ± 23	14 ± 22	12 ± 25	0.170
Proportion of VO_2_ responders (*n*, %)	546 (77%)	99 (86%)	253 (78%)	194 (72%)	0.010 ^b^
% predicted peak VO_2_ (Wasserman-Hansen)	83 ± 22	74 ± 22	84 ± 24	86 ± 18	<0.001^a, b^
% predicted peak VO_2_ (FRIEND-registry)	86 ± 37	77 ± 26	88 ± 44	87 ± 30	0.017^a, b^

*Age groups: younger adults 20–49 yrs; Midlife adults 50–64 yrs; Older adults ≥65 yrs. Continuous data presented as mean ± SD and categorical data presented as n (%).*

*Post-hoc comparisons with Bonferroni correction for multiple comparisons, reveal significant differences (p <0.05) between ^a^young and midlife adults; ^b^young and older adults; and ^c^midlife and older adults.*

*CPET, cardiopulmonary exercise test; HR, heart rate; SBP, systolic blood pressure; DBP, diastolic blood pressure; RPE, rating of perceived exertion; RER, respiratory exchange ratio; METs, metabolic equivalent; VO_2_, oxygen uptake*.

**Table 3:** Exercise testing variables by age group—non-surgical patients.

**Outcome variable**	**Total** **(*n* = 534)**	**Younger adults** **(*n* = 74)**	**Midlife adults** **(*n* = 241)**	**Older adults** **(*n* = 219)**	***p*-value**
**Baseline CPET variables**					
CPET timing prior to program commencement (weeks)	2.2 ± 9.0	3.4 ± 10.9	2.3 ± 9.1	1.7 ± 8.1	0.358
Resting HR (beat.min^−1^)	67 ± 11	71 ± 13	68 ± 10	66 ± 10	0.002^b^
Resting SBP (mmHg)	117 ± 19	108 ± 17	114 ± 17	123 ± 19	<0.001^a, b, c^
Resting DBP (mmHg)	71 ± 10	71 ± 10	72 ± 10	70 ± 10	0.021^c^
Peak exercise HR (beat.min^−1^)	130 ± 22	143 ± 24	135 ± 20	121 ± 18	<0.001^a, b, c^
Peak exercise SBP (mmHg)	163 ± 29	155 ± 32	165 ± 30	162 ± 27	0.032^a^
Peak exercise DBP (mmHg)	67 ± 16	65 ± 16	68 ± 17	66 ± 15	0.200
Peak exercise RPE	18.2 ± 0.8	18.1 ± 0.7	18.2 ± 0.9	18.2 ± 0.8	0.411
Peak exercise RER	1.19 ± 0.09	1.20 ± 0.11	1.20 ± 0.09	1.9 ± 0.10	0.251
Peak workload (estimated METs)	7.1 ± 1.5	7.6 ± 1.6	7.4 ± 1.5	6.5 ± 1.35	<0.001^b, c^
Peak absolute VO_2_ (L.min^−1^)	1.90 ± 0.62	2.18 ± 0.69	2.10 ± 0.61	1.59 ± 0.45	<0.001^b, c^
Peak relative VO_2_ (mL.kg.min^−1^)	21.3 ± 6.2	23.7 ± 7.2	22.9 ± 6.4	18.7 ± 4.5	<0.001^b, c^
% predicted peak VO_2_ (Wasserman-Hansen)	81 ± 18	72 ± 16	83 ± 20	82 ± 17	<0.001^a, b^
% predicted peak VO_2_ (FRIEND-registry)	86 ± 31	78 ± 23	90 ± 37	84 ± 26	0.009^a^
Peak oxygen pulse (mL.beat^−1^)	14.6 ± 4.0	15.2 ± 4.4	15.6 ± 3.8	13.2 ± 3.7	<0.001^b, c^
**Post CPET and CR program variables**					
CPET timing following program commencement (weeks)	15.7 ± 7.5	14.5 ± 8.3	16.2 ± 8.2	15.5 ± 6.3	0.071
CR program duration (weeks)	16.0 ± 7.3	14.6 ± 7.9	16.7 ± 8.1	15.8 ± 5.9	0.072
CR program exercise sessions	29 ± 12	27 ± 14	28 ± 13	31 ± 10	<0.001^b, c^
Delta change in peak relative VO_2_ (mL.kg.min^−1^)	2.2 ± 3.3	3.4 ± 3.9	2.5 ± 3.5	1.5 ± 2.7	<0.001^b, c^
Percent change in peak relative VO_2_ (%)	11 ± 19	16 ± 18	12 ± 18	10 ± 20	0.043^b^
Delta change in peak workload (METs)	0.6 ± 1.0	0.8 ± 1.1	0.6 ± 1.0	0.6 ± 1.0	0.280
Percent change in peak workload (%)	11 ± 19	13 ± 17	10 ± 17	12 ± 22	0.483
Proportion of VO_2_ responders (n, %)	416 (78%)	64 (86%)	191 (79%)	161 (74%)	0.053
% predicted peak VO_2_ (Wasserman-Hansen)	89 ± 20	82 ± 19	91 ± 22	89 ± 17	0.007^a^
% predicted peak VO_2_ (FRIEND-registry)	92 ± 38	88 ± 24	96 ± 46	90 ± 31	0.085

*Age groups: younger adults 20–49 yrs; Midlife adults 50–64 yrs; Older adults ≥65 yrs. Continuous data presented as mean ± SD and categorical data presented as n (%).*

*Post-hoc comparisons with Bonferroni correction for multiple comparisons, reveal significant differences (p <0.05) between ^a^young and midlife adults; ^b^young and older adults; and ^c^midlife and older adults.*

*CPET, cardiopulmonary exercise test; HR, heart rate; SBP, systolic blood pressure; DBP, diastolic blood pressure; RPE, rating of perceived exertion; RER, respiratory exchange ratio; METs, metabolic equivalent; VO_2_, oxygen uptake*.

### Post Exercise Testing and CR Program Outcomes

**Figure 1:**
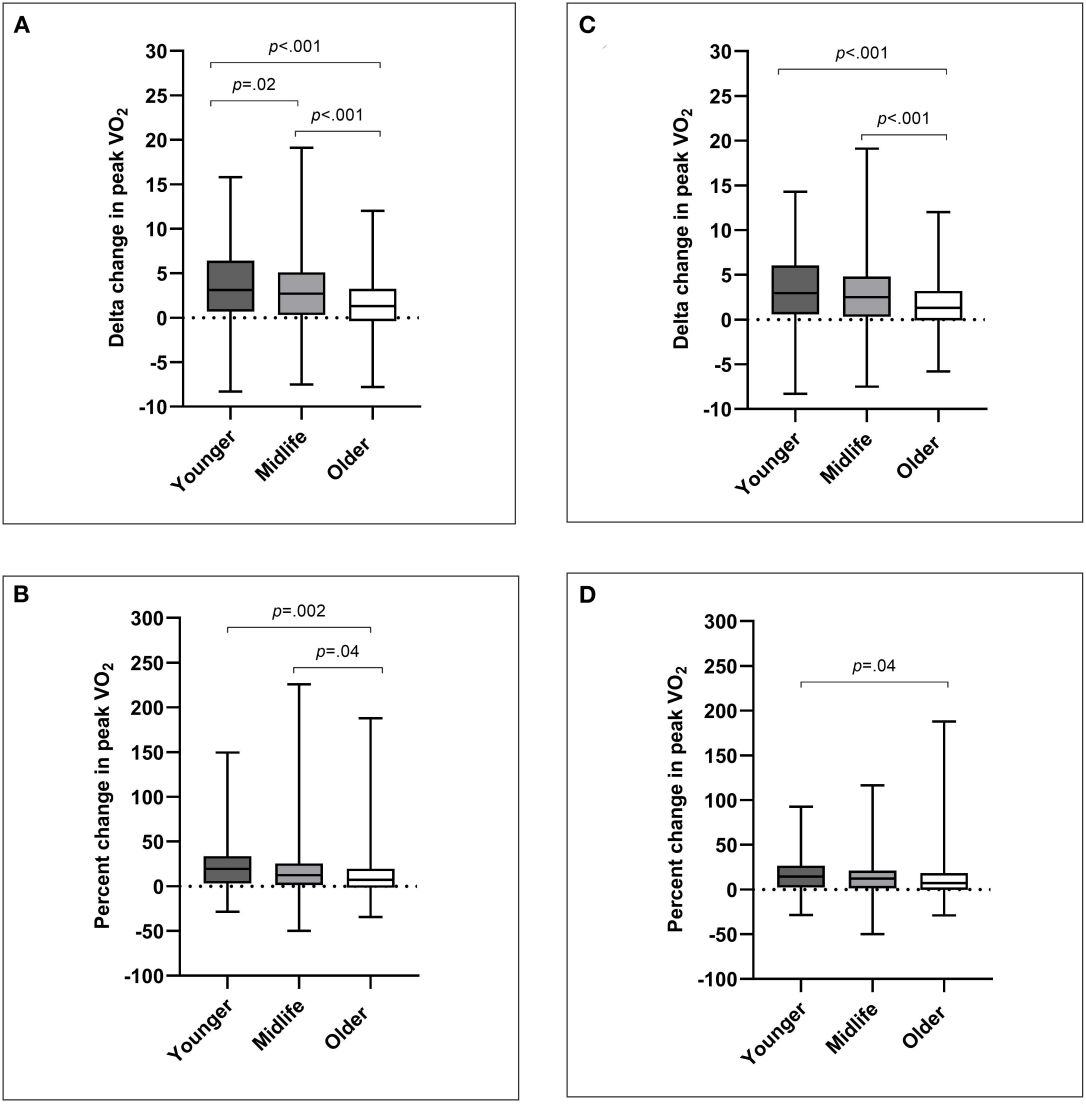
Age-related differences in cardiorespiratory fitness as: **(A)** delta change in peak VO_2_ for all CR indications; **(B)** percent change in peak VO_2_ for all CR indications; **(C)** delta change in peak VO_2_ for non-surgical CR indications; **(D)** percent change in peak VO_2_ for non-surgical CR indications. Data are presented as box and whisker plots with median line, and minimum to maximum.

CR program variables and outcomes for percentage of predicted VO_2_, change in peak VO_2_, change in peak workload (in estimated METs), and proportion of peak VO_2_ responders are presented by age group in Table 2 and age decade in Supplementary Table 2. On average, patients improved relative peak VO_2_ from pre to post CR by 2.5 ± 3.7 mL.kg.min^−1^ (*p* < 0.001), with an average percent change in relative peak VO_2_ of 16 ± 28% (*p* < 0.001). All age groups significantly improved peak VO_2_ (*p* < 0.001) from pre to post CR (Table 2). However, the change in relative peak VO_2_ was lower with increasing age, with significant differences between younger and midlife adults (mean difference = 0.9 mL.kg.min^−1^; *p* = 0.02), younger and older adults (mean difference = 2.1 mL.kg.min^−1^; *p* < 0.001), and midlife and older adults (mean difference 1.2 mL.kg.min^−1^; *p* < 0.001) (Figure 1A). Percent change in relative peak VO_2_ was also lower with increasing age, although differences between groups were only significant for younger and older adults (mean difference = 11%; *p* = 0.002), and midlife and older adults (mean difference = 5%; *p* = 0.04) (Figure 1B). On average, 77% of patients showed an improvement in peak VO_2_ (>0% change) from pre to post cardiac rehabilitation. While response rate showed a lower trend with increasing age, the response rate was only significantly different between younger and older adults (86 vs. 72%, respectively; *p* = 0.008). Change in peak workload was lower for older adults compared with younger adults (0.6 ± 1.1 vs. 1.0 ± 1.2 METs; *p* = 0.007), however percent change in peak workload was not different between groups. On average, there was an improvement in percentage of predicted VO_2_ from 75 ± 22% to 83 ± 22%, however levels remained significantly lower for younger adults compared with midlife adults (74 ± 22 vs. 84 ± 24; *p* < 0.001) and older adults (74 ± 22 vs. 86 ± 18; *p* < 0.001). Older adults completed a significantly greater number of CR sessions than younger adults (30 ± 11 vs. 24 ± 14 sessions; *p* < 0.001) and midlife adults (30 ± 11 vs. 26 ± 13 sessions; *p* < 0.001), and older adults were in the program for a longer period than younger adults (17 ± 9 vs. 13 ± 9 weeks; *p* = 0.001). The post CPET occurred on average 15.9 ± 9.3 weeks following CR commencement, and within 0.4 ± 11.5 weeks of CR completion.

For sensitivity analyses excluding surgical patients (Table 3), the average improvement in relative peak VO_2_ was moderately lower (2.2 ± 3.3 mL.kg.min^−1^) than the primary analysis, as well as the average percent change in relative peak VO_2_ (11 ± 20%). Change in relative peak VO_2_ remained lower for older adults compared with younger adults (mean difference = 1.9 mL.kg.min^−1^; *p* < 0.001) and midlife adults (mean difference = 1.0 mL.kg.min^−1^; *p* < 0.001) (Figure 1C). Differences in the percent change of relative VO_2_ were less pronounced between older and younger adults (mean difference = 6%; *p* = 0.04), and there were no longer differences between younger and midlife adults or midlife and older adults (Figure 1D). There was no longer an effect of age group for proportion of VO_2_ responders. Moreover, percentage change in peak workload was not different between age groups.

In Supplementary Table 3, we display changes in peak VO_2_ for specific CR indications such as acute coronary syndrome (ACS), PCI, heart transplant, and coronary artery bypass graft (CABG)/valve surgery. Numerically, the ACS sub-group showed a similar trend to our non-surgical cohort. In the PCI sub-group, younger adults show a considerably higher delta and percent change in peak VO_2_ when compared to both midlife and older adults. In the CABG/valve and other sub-groups, delta and percent change in peak VO_2_ are markedly reduced in older adults compared with younger and midlife adults. In the heart transplant sub-group, all age groups showed dramatically higher delta and percent change in peak VO_2_ compared with other CR indications, however in contrast to other CR indications, older adults showed markedly higher improvements in peak VO_2_ than younger and midlife adults.

We did not find an association of female sex for delta and percent change in peak VO_2_, respectively, in younger adults [−0.7 (−2.3 to 0.8) ml.kg.min^−1^; *p* = 0.34 and 7.4 (−3.3 to 18.1) %; *p* = 0.17] or older adults [−0.2 (−1.1 to 0.7) ml.kg.min^−1^; *p* = 0.71; and 0.1 (−7.6 to 7.7) %; *p* = 0.98]. There was trend toward an influence of female sex in midlife adults for delta change in peak VO_2_ [−1.0 (−2.0 to 0.0) ml.kg.min^−1^; *p* = 0.05] but not percent change in peak VO_2_ [−3.1 (−10.7 to 4.5) %; *p* = 0.42]. There was also no influence of female sex on likelihood of peak VO_2_ response for younger adults [0.6 (0.2–1.9); *p* = 0.42], midlife adults [1.1 (0.6–2.1); *p* = 0.82], or older adults [1.2 (0.6–2.3); *p* = 0.57].

## Discussion

This study investigated age-related differences for improving cardiorespiratory fitness (peak VO_2_) and exercise capacity (as peak workload) during a CR program. All age groups significantly improved peak VO_2_ from pre to post CR, however when evaluating patients with any CR indication, we found significant attenuation in peak VO_2_ improvement with increasing age, from younger adults to midlife adults, and midlife adults to older adults. Although older adults improved peak VO_2_, their percent change in peak VO_2_ was significantly lower when compared with both younger adults and midlife adults; and older adults were less likely to show a peak VO_2_ response (>0% change) to the CR program compared with younger adults. When surgical patients were excluded from the analysis, age-related differences for percent change in peak VO_2_ were reduced, however older adults still achieved a lower improvement in cardiorespiratory fitness during CR. Percent change in peak exercise workload showed no age-related differences throughout CR.

Previous research in healthy populations has shown that cardiorespiratory fitness decreases by approximately 1-MET (equivalent to 3.5 ml.kg.min^−1^) with each increasing age decade (10, 34). In the current study, the differences between age-decades using directly measured peak VO_2_ at the beginning of CR were much less pronounced (mean difference ~ 1.0 ml.kg.min^−1^). Our results were similar to the differences between age-decades reported by Banks et al. (16) (mean difference in peak VO_2_ = 1.8 ml.kg.min^−1^) in their patients with coronary artery disease undergoing CR. With regards to changes in peak VO_2_ with exercise training, our findings were also similar to Banks et al., with older patients attending CR showing both lower delta and percent change in peak VO_2_. These results are in contrast to studies in healthy populations showing similar percent change in peak VO_2_ between age groups with exercise training (12–14). The Heritage Family Study investigated the variability in peak VO_2_ response to a standardized exercise training program in a healthy population. They found that while older adults had a lower delta change in relative peak VO_2_, the percent change from baseline was similar to younger adults (12). Robinson et al. (35) also found that healthy younger and older adults had similar percent change in peak VO_2_ with combined training (aerobic + resistance). In the same study, both young and older adults showed significant improvements in peak VO_2_ with high intensity interval training, however, the percent change was much greater in the younger adults (35). Therefore, differences in training intensity could have influenced our age-related differences in peak VO_2_ improvement.

Currently there are limited published data showing the influence of peak VO_2_ improvement during CR, as a percent change from baseline, on major adverse cardiovascular events. However, the differences in delta change for relative peak VO_2_ that we found between our age groups could be considered clinically meaningful, as each 1 ml.kg.min^−1^ increase in peak VO_2_ during cardiac rehabilitation has been shown to reduce cardiovascular events by 21% and all-cause mortality by 13% (8). Recent work by Carbone et al. (36) found that the percentage of predicted peak VO_2_ at the end of CR was a stronger predictor of long-term survival rather than change in peak VO_2_ during CR. While our results showed younger adults had greater improvements in peak VO_2_ during CR, midlife and older adults maintained a significantly higher percentage of predicted peak VO_2_ at the end of CR, compared with the younger adults. A potential bias with this outcome is that older adults who are able to complete a CPET pre and post CR could be more likely to have a higher fitness level and may not be representative of the average older adult attending CR.

Notably, our midlife and older adults also achieved a significantly higher percentage of predicted peak VO_2_ at the beginning of CR (~76–80%), compared with our younger adults (~63%). Lower baseline peak VO_2_ has consistently been shown to be a strong predictor for peak VO_2_ response with exercise training (37, 38). Although mean baseline peak VO_2_ was lower for older adults, the mean percentage of predicted peak VO_2_ was lower for younger adults, and this likely contributed to the greater improvements in peak VO_2_ during CR. The lower initial fitness level in our younger adults was in part related to the increased prevalence of heart transplant. Indeed, when we excluded surgical patients from the analysis, the mean relative peak VO_2_ at baseline increased by 2.7 ml.kg.min^−1^ (13%) in our younger adults and 1.8 ml.kg.min^−1^ (9%) in our midlife adults, with minimal change in our older adults (0.5 ml.kg.min^−1^; 3%). In non-surgical patients, the age-related differences between younger and midlife adults diminished. Therefore, differences in patient populations among age groups may contribute to bias that CR is less effective for improving peak VO_2_ with increasing age. Previous research has shown that females have a lower improvement in peak VO_2_ than males during CR (39). We found a trend toward female adults in midlife achieving a lower delta change for peak VO_2_ improvement, however, there was no significant influence of sex on peak VO_2_ improvement or likelihood for peak VO_2_ response in any age group.

Multimorbidity may have contributed to the lower improvement in peak VO_2_ in our older adults. Having a higher number of comorbidities has been associated with lower improvements in cardiorespiratory fitness (40) and other CR outcomes (41). Although our older adults attended more sessions and were in the program for longer, they also had a higher prevalence of peripheral artery disease, stroke, myocardial infarction, and chronic obstructive pulmonary disease. This is consistent with the work of Listerman et al. (41), showing that patients with a higher number of comorbidities may be prescribed a greater number of CR sessions than patients with a lower number of comorbidities.

Our study has a number of limitations that warrant discussion. Firstly, patients were from a single-center, were predominately male (75%), and the majority of the population in this region (Olmsted County) are White. Secondly, the retrospective nature of this study can present several sources of bias, and only including patients with pre- and post-CR CPET data may contribute to selection bias. These factors might affect the generalizability, but not the internal validity of our findings. Medication differences between age groups could not be accounted for in our ANOVA *post-hoc* analyses. Using an Analysis of Covariance (ANCOVA), we explored the interactions between medication status and change in peak VO_2_, in this case we only found an interaction between delta change in peak VO_2_ and angiotensin-converting enzyme or angiotensin II receptor blocker. When including this medication status as a covariate with ANCOVA, the effect of age group on delta change in peak VO_2_ remained significant (*p* < 0.001). Similarly, differences in baseline CPET timing between groups could not be accounted for in our ANOVA *post-hoc* analyses. Using ANCOVA, an interaction was found between baseline CPET timing and percent change in peak VO_2_, but not delta change in peak VO_2_. When including baseline CPET timing as a covariate with ANCOVA, the effect of age group on percent change in peak VO_2_ remained significant (*p* < 0.001). Moreover, when surgical patients were excluded, baseline CPET timing was not different between age groups. Likewise, although informative, we had limited sample size to evaluate statistical significance between age groups for specific CR indications (e.g., ACS, PCI, CABG, heart transplant). Finally, due to the absence of exercise prescription data during the cardiac rehabilitation program, we could not evaluate whether the greater improvement in peak VO_2_ of younger adults was influenced by exercise training being of a higher relative intensity or longer duration.

## Conclusion

This study found significant improvements in peak VO_2_ for all age groups during a CR program. However, there was a significant attenuation in both delta and percent change in peak VO_2_, from younger to midlife adults, and midlife to older adults with any CR referral diagnosis. Older adults also showed a significantly lower rate of VO_2_ response to a CR program compared with younger adults. Age-related differences were influenced by a higher prevalence of heart transplant in our younger age group. In non-surgical patients, age-related differences in percent change of peak VO_2_ were less pronounced, however older adults still had lower peak VO_2_ improvement during CR. These findings may have implications for individualizing CR programming based on age and comorbid conditions.

## Data Availability Statement

The original contributions presented in the study are included in the article/Supplementary Material, further inquiries can be directed to the corresponding author/s.

## Ethics Statement

The studies involving human participants were reviewed and approved by Mayo Clinic Institutional Review Board Olmsted Medical Center Institutional Review Board. Written informed consent for participation was not required for this study in accordance with the national legislation and the institutional requirements.

## Author Contributions

JT, JM-I, AC-S, JS, RS, RT, BJ, TO, and AB contributed to study conception and design. JT, JM-I, AC-S, and JS contributed to data collection and analysis. JT and JM-I contributed to the statistical analysis. JT drafted the manuscript. JM-I, AC-S, JS, RS, RT, BJ, TO, and AB critically reviewed the manuscript. All authors contributed to the article and approved the submitted version.

## Funding

This study used the resources of the Rochester Epidemiology Project (REP) medical records-linkage system, which was supported by the National Institute on Aging (NIA; AG 058738), by the Mayo Clinic Research Committee, and by fees paid annually by REP users, as well as the National Institutes of Nursing Research R01NR018832 (TO).

## Author Disclaimer

The content of this article is solely the responsibility of the authors and does not represent the official views of the National Institutes of Health (NIH) or the Mayo Clinic.

## Conflict of Interest

The authors declare that the research was conducted in the absence of any commercial or financial relationships that could be construed as a potential conflict of interest.

## Publisher's Note

All claims expressed in this article are solely those of the authors and do not necessarily represent those of their affiliated organizations, or those of the publisher, the editors and the reviewers. Any product that may be evaluated in this article, or claim that may be made by its manufacturer, is not guaranteed or endorsed by the publisher.
