# Supplementary material for: Age-Related Differences for Cardiorespiratory Fitness Improvement in Patients Undergoing Cardiac Rehabilitation
Source: Front Cardiovasc Med. 2022 Apr 14;9:872757. doi: 10.3389/fcvm.2022.872757 (PMC9047908; doi:10.3389/fcvm.2022.872757)
Supplement: Supplementary file 1 [file Table_1.DOCX]

**Supplementary Digital Content**

Age-related Differences for Cardiorespiratory Fitness Improvement in Patients Undergoing Cardiac Rehabilitation

**Table S1:** Patient Demographics by Age Decade

**Table S2:** Exercise Testing Variables by Age Decade

**Table S3**: Baseline Peak VO_2_ and Changes in Peak VO_2_ for Specific Cardiac Rehabilitation Indications

Table S1: Patient Demographics by Age Decade

| **Demographic variable**  **(Total n = 708)** | **<40yrs (n=41)** | **40-49yrs (n=74)** | **50-59yrs (n=207)** | **60-69rs (n=221)** | **70-79yrs (n=141)** | **80-89yrs (n=24)** | **p value** | |
| --- | --- | --- | --- | --- | --- | --- | --- | --- |
| Age (years) | 32.4 ± 5.1 | 46.0 ± 2.8 | 55.5 ± 2.8 | 65.0 ± 2.7 | 74.7 ± 2.8 | 82.3 ± 1.9 | < 0.001 | |
| Female | 14 (34%) | 26 (35%) | 46 (22%) | 46 (21%) | 27 (19%) | 8 (33%) | 0.035 | |
| Body mass index (kg.m^-2^) | 29.8 ± 7.4 | 29.1 ± 5.5 | 30.2 ± 5.4 | 29.4 ± 5.3 | 29.2 ± 4.3 | 26.9 ± 4.0 | 0.067 | |
| Rehab duration (weeks) | 11.3 (6.5) | 13.8 (10.1) | 15.2 (11.4) | 15.7 (10.1) | 17.3 (8.7) | 18.2 (7.4) | 0.007 | |
| Rehab Sessions | 22.6 (11.5) | 24.7 (14.5) | 24.9 (13.7) | 27.8 (12.2) | 30.9 (10.8) | 30.4 (8.7) | < 0.001 | |
| **Indication for cardiac rehabilitation** | | | | | | | |  |
| Acute coronary syndrome | 5 (12%) | 22 (30%) | 58 (28%) | 57 (26%) | 49 (35%) | 5 (21%) | 0.089 | |
| PCI | 4 (10%) | 14 (19%) | 73 (35%) | 68 (31%) | 53 (38%) | 11 (46%) | < 0.001 | |
| Surgical | 18 (44%) | 23 (31%) | 51 (25%) | 58 (26%) | 20 (14%) | 3 (12%) | 0.001 | |
| Heart transplant | 14 (34%) | 16 (22%) | 40 (19%) | 27 (12%) | 3 (2%) | 0 (0%) | < 0.001 | |
| CABG | 2 (5%) | 2 (3%) | 7 (3%) | 14 (6%) | 8 (6%) | 3 (12%) | 0.34 | |
| Valve | 2 (5%) | 4 (5%) | 3 (1%) | 15 (7%) | 9 (6%) | 0 (0%) | 0.099 | |
| Other ‡ | 14 (34%) | 15 (20%) | 25 (12%) | 37 (17%) | 19 (13%) | 5 (21%) | 0.013 | |
| **Co-morbidities** |  |  |  |  |  |  |  | |
| Myocardial infarction | 7 (17%) | 25 (34%) | 72 (35%) | 81 (37%) | 63 (45%) | 12 (50%) | 0.023 | |
| Hypertension | 15 (37%) | 46 (62%) | 160 (77%) | 185 (84%) | 130 (92%) | 24 (100%) | < 0.001 | |
| Chronic heart failure | 23 (56%) | 25 (34%) | 68 (33%) | 76 (34%) | 46 (33%) | 10 (42%) | 0.096 | |
| Dyslipidemia | 23 (56%) | 60 (81%) | 193 (93%) | 206 (93%) | 136 (96%) | 24 (100%) | < 0.001 | |
| Arrhythmia | 28 (68%) | 50 (68%) | 163 (79%) | 181 (82%) | 118 (84%) | 21 (88%) | 0.025 | |
| Stroke | 3 (7%) | 12 (16%) | 34 (16%) | 46 (21%) | 48 (34%) | 10 (42%) | < 0.001 | |
| Chronic kidney disease | 9 (22%) | 18 (24%) | 65 (31%) | 68 (31%) | 47 (33%) | 10 (42%) | 0.446 | |
| COPD | 3 (7%) | 28 (38%) | 83 (40%) | 102 (46%) | 63 (45%) | 9 (38%) | < 0.001 | |
| Diabetes | 11 (27%) | 34 (46%) | 123 (59%) | 145 (66%) | 95 (67%) | 15 (62%) | < 0.001 | |
| Peripheral artery disease | 0 (0%) | 5 (7%) | 23 (11%) | 34 (15%) | 23 (16%) | 12 (50%) | < 0.001 | |
| Smoking | 22 (54%) | 42 (57%) | 128 (62%) | 153 (69%) | 94 (67%) | 12 (50%) | 0.107 | |
| **Medications** |  |  |  |  |  |  |  | |
| β-blocker | 20 (49%) | 60 (81%) | 155 (75%) | 173 (78%) | 122 (87%) | 20 (83%) | < 0.001 | |
| ACE Inhibitor or AR2B | 16 (39%) | 35 (47%) | 107 (52%) | 125 (57%) | 89 (63%) | 17 (71%) | 0.021 | |
| Calcium channel blocker | 6 (15%) | 17 (23%) | 45 (22%) | 64 (29%) | 38 (27%) | 9 (38%) | 0.172 | |
| Diuretics | 14 (34%) | 25 (34%) | 75 (36%) | 100 (45%) | 78 (55%) | 13 (54%) | 0.003 | |
| Salicylates or anti-platelet | 14 (34%) | 52 (70%) | 144 (70%) | 156 (71%) | 113 (80%) | 22 (92%) | < 0.001 | |
| Anticoagulant | 8 (20%) | 19 (26%) | 29 (14%) | 42 (19%) | 35 (25%) | 6 (25%) | 0.119 | |
| Cholesterol lowering | 14 (34%) | 55 (74%) | 172 (83%) | 180 (81%) | 123 (87%) | 22 (92%) | < 0.001 | |

Continuous data are presented as mean ± SD and categorical data presented as n (%).

Abbreviations: PCI – percutaneous coronary intervention; CABG – coronary artery bypass graft surgery; COPD – chronic obstructive pulmonary disease; ACE – angiotensin-converting enzyme; ARB – angiotensin II receptor blocker. ‡ Breakdown of other category includes stable angina (4%), heart failure (2%), dyspnea (<1%), sudden cardiac death (<1%), peripheral artery disease (<1%), pericarditis or myocarditis (<1%), or non-specified cardiac event (8%).

Table S2: Exercise Testing Variables by Age Decade

| **Outcome variable**  **(Total n = 708)** | **<40yrs**  **(n=41)** | | **40-49yrs (n=74)** | **50-59yrs (n=207)** | **60-69rs (n=221)** | **70-79yrs (n=141)** | | | **80-89yrs (n=24)** | | | **p value** | | | |  |  |
| --- | --- | --- | --- | --- | --- | --- | --- | --- | --- | --- | --- | --- | --- | --- | --- | --- | --- |
| **Baseline CPET variables** | | | |  |  | |  | | |  | | | |  | | | |
| Resting HR (beat.min^-1^) | 74 ± 13 | 71 ± 13 | | 70 ± 11 | 68 ± 11 | 66 ± 11 | | 63 ± 8 | | | < 0.001 | | | |  |  |  |
| Resting SBP (mmHg) | 101 ± 19 | 108 ± 17 | | 112 ± 18 | 116 ± 19 | 123 ± 20 | | 122 ± 20 | | | < 0.001 | | | |  |  |  |
| Resting DBP (mmHg) | 65 ± 15 | 70 ± 15 | | 72 ± 12 | 71 ± 10 | 70 ± 10 | | 63 ± 13 | | | 0.001 | | | |  |  |  |
| Peak exercise HR (beat.min^-1^) | 139 ± 32 | 133 ± 24 | | 131 ± 24 | 124 ± 21 | 119 ± 18 | | 115 ± 17 | | | < 0.001 | | | |  |  |  |
| Peak exercise SBP (mmHg) | 131 ± 37 | 147 ± 35 | | 155 ± 40 | 155 ± 33 | 159 ± 31 | | 151 ± 19 | | | < 0.001 | | | |  |  |  |
| Peak exercise DBP (mmHg) | 57 ± 16 | 65 ± 19 | | 65 ± 20 | 68 ± 13 | 67 ± 14 | | 54 ± 24 | | | < 0.001 | | | |  |  |  |
| Peak exercise RPE | 18.1 ± 0.9 | 18.2 ± 0.7 | | 18.2 ± 1.0 | 18.2 ± 0.8 | 18.2 ± 0.8 | | 18.0 ± 1.0 | | | 0.838 | | | |  |  |  |
| Peak exercise RER | 1.2 ± 0.1 | 1.2 ± 0.1 | | 1.2 ± 0.1 | 1.2 ± 0.1 | 1.2 ± 0.1 | | 1.2 ± 0.1 | | | 0.308 | | | |  |  |  |
| Peak workload (estimated METs) | 6.9 ± 2.2 | 7.0 ± 1.6 | | 7.2 ± 1.8 | 6.7 ± 1.5 | 6.4 ± 1.4 | | 6.0 ± 1.5 | | | < 0.001 | | | |  |  |  |
| Peak absolute VO_2_ (L.min^-1^) | 1.87 ± 0.90 | 1.84 ± 0.73 | | 1.98 ± 0.74 | 1.71 ± 0.56 | 1.54 ± 0.41 | | 1.22 ± 0.32 | | | < 0.001 | | | |  |  |  |
| Peak relative VO_2_ (mL.kg.min^-1^) | 20.8 ± 9.3 | 21.1 ± 7.5 | | 21.6 ± 7.4 | 19.6 ± 6.2 | 18.1 ± 4.3 | | 16.4 ± 3.5 | | | < 0.001 | | | |  |  |  |
| % predicted peak VO_2_ (Wasserman-Hansen) | 56 ± 21 | 67 ± 20 | | 76 ± 24 | 76 ± 22 | 81 ± 17 | | 83 ± 15 | | | < 0.001 | | | |  |  |  |
| % predicted peak VO_2_ (FRIEND-registry) | 60 ± 25 | 71 ± 26 | | 80 ± 29 | 82 ± 40 | 83 ± 28 | | 81 ± 22 | | | < 0.001 | | | |  |  |  |
| Peak oxygen pulse (mL.beat^-1^) | 13.0 ± 5.2 | 13.6 ± 4.6 | | 14.8 ± 4.5 | 13.8 ± 4.1 | 13.1 ± 3.5 | | 10.7 ± 2.5 | | | < 0.001 | | | |  |  |  |
| **Post CPET variables following cardiac rehabilitation** | | | | | | | | | | | | | | | | | |
| % predicted peak VO_2_ (Wasserman-Hansen) | 66 ± 19 | 79 ± 22 | | 84 ± 24 | 85 ± 22 | 86 ± 17 | | 89 ± 18 | | | | | < 0.001 | | | |  |
| % predicted peak VO_2_ (FRIEND-registry) | 69 ± 22 | 82 ± 27 | | 87 ± 27 | 89 ± 49 | 88 ± 35 | | 84 ± 23 | | | | | 0.037 | | | |  |
| Delta change in peak relative VO_2_ (mL.kg.min^-1^) | 3.5 ± 4.4 | 3.8 ± 3.8 | | 2.7 ± 3.8 | 2.5 ± 3.8 | 1.4 ± 3.0 | | 1.0 ± 2.4 | | | | | < 0.001 | | | |  |
| Percent change in peak relative VO_2_ (%) | 26 ± 33 | 21 ± 24 | | 17 ± 28 | 18 ± 32 | 9 ± 20 | | 6 ± 15 | | | | | 0.001* | | | |  |
| Delta change in peak workload (METs) | 0.9 ± 1.2 | 1.0 ± 1.2 | | 0.7 ± 1.1 | 0.7 ± 1.1 | 0.6 ± 1.1 | | 0.4 ± 1.3 | | | | | 0.126 | | | |  |
| Percent change in peak workload (%) | 18 ± 26 | 17 ± 22 | | 13 ± 21 | 14 ± 27 | 11 ± 21 | | 11 ± 25 | | | | | 0.545 | | | |  |
| Proportion of VO_2_ responders (n, %) | 34 (83%) | 65 (88%) | | 161 (78%) | 170 (77%) | 101 (72%) | | 15 (62%) | | | | | 0.052 | | | |  |

Continuous data are presented as mean ± SD and categorical data presented as n (%).

*Post-hoc comparisons revealed significant differences (p<0.05) between <40yrs group and all other age groups.

Abbreviations: CPET – cardiopulmonary exercise test; HR – heart rate; SBP – systolic blood pressure; DBP – diastolic blood pressure; RPE – rating of perceived exertion; RER – respiratory exchange ratio; METs – metabolic equivalent; VO_2_ – oxygen uptake

Table S3: Baseline Peak VO_2_ and Changes in Peak VO_2_ for Specific Cardiac Rehabilitation Indications

| **Outcome variable** | **Total** | **Younger adults** | | **Midlife adults** | **Older adults** | |
| --- | --- | --- | --- | --- | --- | --- |
| **Total cohort** | **n=708** | **n=115** | | **n=324** | **n=269** | |
| Peak relative VO_2_ (mL.kg.min^-1^) | 20.0 ± 6.7 | 21.0 ± 8.1 | | 21.1 ± 7.1 | 18.2 ± 4.9 | |
| Delta change in peak relative VO_2_ (mL.kg.min^-1^) | 2.5 ± 3.7 | 3.7 ± 4.0 | | 2.8 ± 3.8 | 1.6 ± 3.2 | |
| Percent change in peak relative VO_2_ (%) | 16 ± 28 | 23 ± 28 | | 17 ± 28 | 12 ± 27 | |
| **Acute coronary syndrome** | **n=196** | **n=27** | | **n=90** | **n=79** | |
| Peak relative VO_2_ (mL.kg.min^-1^) | 22.0 ± 6.7 | 25.6 ± 6.9 | | 24.8 ± 6.2 | 18.9 ± 4.4 | |
| Delta change in peak relative VO_2_ (mL.kg.min^-1^) | 2.3 ± 3.3 | 4.3 ± 4.3 | 2.6 ± 3.3 | | 1.3 ± 2.4 |  |
| Percent change in peak relative VO_2_ (%) | 10 ± 14 | 17 ± 16 | 11 ± 13 | | 7 ± 13 |  |
| **Percutaneous coronary intervention** | **n=223** | **n=18** | **n=105** | | **n=100** |  |
| Peak relative VO_2_ (mL.kg.min^-1^) | 21.5 ± 5.9 | 26.4 ± 5.8 | 22.9 ± 6.2 | | 19.2 ± 4.5 |  |
| Delta change in peak relative VO_2_ (mL.kg.min^-1^) | 2.5 ± 3.3 | 4.4 ± 3.4 | 2.5 ± 3.6 | | 2.0 ± 2.7 |  |
| Percent change in peak relative VO_2_ (%) | 13 ± 18 | 18 ± 15 | 12 ± 18 | | 12 ± 18 |  |
| **Heart transplant** | **n=99** | **n=30** | **n=55** | | **n=14** |  |
| Peak relative VO_2_ (mL.kg.min^-1^) | 12.8 ± 4.1 | 12.8 ± 4.2 | 13.1 ± 4.3 | | 11.3 ± 2.6 |  |
| Delta change in peak relative VO_2_ (mL.kg.min^-1^) | 5.0 ± 4.1 | 4.9 ± 3.7 | 4.7 ± 4.4 | | 6.7 ± 6.2 |  |
| Percent change in peak relative VO_2_ (%) | 48 ± 44 | 44 ± 37 | 45 ± 46 | | 68 ± 48 |  |
| **CABG / Valve** | **n=74** | **n=11** | **n=27** | | **n=36** |  |
| Peak relative VO_2_ (mL.kg.min^-1^) | 20.2 ± 6.7 | 24.8 ± 7.6 | 21.3 ± 6.6 | | 18.0 ± 5.7 |  |
| Delta change in peak relative VO_2_ (mL.kg.min^-1^) | 1.1 ± 4.3 | 3.0 ± 5.4 | 1.5 ± 4.3 | | 0.2 ± 3.9 |  |
| Percent change in peak relative VO_2_ (%) | 8 ± 26 | 11 ± 21 | 10 ± 25 | | 5 ± 27 |  |
| **Other** | **n=115** | **n=29** | **n=46** | | **n=40** |  |
| Peak relative VO_2_ (mL.kg.min-1) | 18.7 ± 5.8 | 20.3 ± 7.0 | 19.2 ± 5.7 | | 17.1 ± 4.4 |  |
| Delta change in peak relative VO_2_ (mL.kg.min-1) | 1.7 ± 3.4 | 1.9 ± 3.4 | 2.4 ± 3.8 | | 0.8 ± 2.8 |  |
| Percent change in peak relative VO_2_ (%) | 12 ± 27 | 14 ± 22 | 14 ± 25 | | 8 ± 33 |  |

Age groups: Younger adults 20-49yrs; Midlife adults 50-64yrs; Older adults ≥65yrs. Continuous data presented as mean ± SD.

Abbreviations: VO_2_ – oxygen uptake. CABG – coronary artery bypass graft surgery.

‡ Breakdown of other category includes, stable angina (4%), heart failure (2%), dyspnea (<1%), sudden cardiac death (<1%), peripheral artery disease (<1%), pericarditis or myocarditis (<1%), or non-specified cardiac event (8%).
